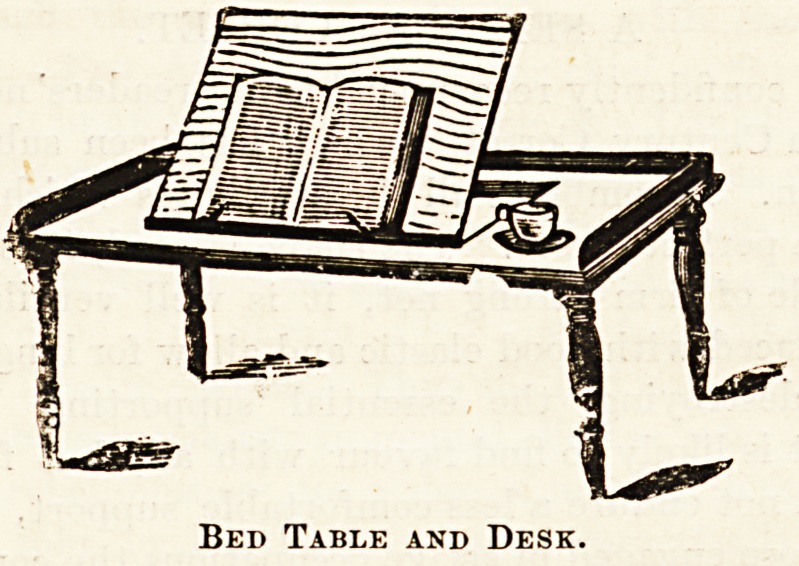# "The Hospital" Nursing Mirror

**Published:** 1899-04-15

**Authors:** 


### "The Hospital" Nursing Mirror.

Being the Nursing Section of "The Hospital."

[Contributions for this Section of "The Hospital" should be addressed to the Editor, The Hospital, 28 & 29, Southampton Street, Strand, London, W.C., and should have the word "Nursing" plainly written in left-hand top corner of the envelope.]

### Notes on News from the Nursing World.

### THE CAFE CHANTANT AT THE HOTEL CECIL.

It is satisfactory to learn that the entertainment in aid of extending the work of the Royal British Nurses' Association is meeting with enthusiastic support. Feeble efforts have been made to disparage the movement, on the stupid ground that it is derogatory to the dignity of members of the nursing profession; but little notice is taken of such ignorant criticisms, for it is well known that the object in view is not to place the association in a sound position with regard to current expenses. The object is to carry out certain developments necessary in the interests of the profession which cannot be attempted until some invested funds are again at the disposal of the corporation; and it is a libel on the Royal president, the patronesses, the many ladies and gentlemen who have promised their services on the occasion, and the nurses themselves, to talk about the fête as "a form of pauperising trained nurses." The Princess Christian, who has always been foremost in recognising the work done by nurses, would be the last person to associate herself with any step likely to injure their professional status, which as a matter of fact is not at stake.

### THE WRECK OF THE "STELLA."

We regret to hear that, though Miss Baker, of Nightingale Lodge, St. Thomas's Hospital, was saved from the wreck of the "Stella," she unhappily lost her father. She was unable to supply details of the disaster, but she has, naturally, terribly sad and bitter memories of the awful time she endured, and she says that the personal strain was so great that she "cannot remember seeing anything of the other passengers."

### ICE CREAMS AND DISTRICT NURSES.

The ice cream season is, in a sense, always with us; but the time is approaching when it will be at its height, and the recent death of a child eleven years old from the effects of eating ice cream prompts us to remind district nurses that they might render important help in the direction of modifying the mischief arising from the consumption of the popular, but unwholesome, commodity. They might, for instance, warn mothers to encourage their children to spend their pennies in harmless sweets instead of on ice creams, and the children themselves that it is much more dangerous when they are hot to eat ices than it is to drink cold water. Of course, it is only by constantly repeating the statemen that children can be brought to realise it; but, as to a very large extent, they have learnt the risk of drinking cold water when they are uncomfortably warm, it is by no means hopeless to endeavour to instruct them concerning the graver perils they run in partaking of ice cream under similar conditions.

### THE MASSAGE SCANDAL.

By slow degrees the so-called massage establishments in the West-end of London are being broken up. The vestry of St. James's may be congratulated upon proving that no alteration in the existing law is necessary to deal with a state of things which has long been a public scandal. An attempt was recently made to induce the Home Secretary to appoint a Royal Commission to inquire into the question, but he very wisely declined to entertain the proposal. Commissions are expensive luxuries, and should only be undertaken when an overwhelming case can be urged on behalf of them. With respect to such houses we hope that punishment will overtake the real offenders. It is not the wretched "masseuses" or "manicurists" who deserve to be most severely treated, but the abominable creatures who trade on their frailty. In the interests of the community at large, and especially in those of the trained masseuses whose services are often so valuable, it is essential that they should be stamped out.

### "HOW SICKNESS IS SPREAD."

The head master of an important school cites a striking illustration of the selfish thoughtlessness with which infectious disease is spread. His school has been suffering from more than one infectious complaint during the past term, and, in order to guard as far as possible against the infection being carried elsewhere, a circular was sent to the parents of all the boys ten days before the holidays pointing out the danger of their sons going about indiscriminately until such time had elapsed as to show that they had not the complaint in their system. In reply, one of the parents wrote to his boy that he could not in the circumstances go home at once, "because of the danger to the other children"; but, by way of compensation, he was told that he should be put in charge of a former servant, "who would take him to a number of places of amusement." This case of undiluted selfishness may be supplemented by a fact which came under our notice the other day. A daily dressmaker who went to fulfil an engagement at a private house in a London suburb was told that the mistress of the house was ill, as it transpired afterwards, from scarlet fever, and that she would be advised of her recovery. In a few days the lady's husband called at the dressmaker's house and informed her that his wife was ready to receive her, "as she was quite well, although her hands had not quite finished peeling."

### RETURN OF NURSES FROM THE NIGER.

The "Accra" arrived in Liverpool on Monday, bringing the three Guy's nurses, Miss Nutt, Miss Clarke, and Miss Powell, who have just completed their year's term of service in the hospitals attached to Captain Lugard's expedition into the Niger country. All three have suffered from repeated attacks of fever, and have had their hands full, as sickness has been rife amongst the white men of whom alone they had charge; the nursing of the black troops being relegated to the non-commissioned officers. Fortunately before leaving, the bill of health greatly improved, and there have been no deaths since that of Captain Baker some weeks ago. The British nurses had charge of two hospitals, one at Jebba, where the greater part of their time was spent, and the other at Lokoja. They left Old Calabar on the 12th and Freetown on the 26th of March. So far no nurses have taken their places, and it is not known who will be appointed. Captain Lugard gave an excellent account of their work to the home authorities.

### PRIZE GIVING AT GUY'S HOSPITAL

THE fine old court-room at Guy's Hospital, transformed by skilful arrangements of plants and seats into a very effective drawing-room, was the scene of a pleasant gathering last week, when medals and prizes were distributed to nurses and probationers by Mrs. Cosmo Bonsor, wife of the treasurer. It was the first occasion of the kind, for medals and prizes for first-year probationers are novelties at Guy's, and special interest therefore attached to the proceedings. Nurses mustered in force, and many friends of the hospital and its workers also responded to Miss Nott-Bower's invitation to witness the event and listen to the excellent music provided by the Students' Glee Club and the Nurses' Choral Society. Of the new prizes, two medals and a set of books, awarded to the probationers gaining the three highest aggregate number of marks in the first year examination, were the gift of members of the Cazenove family in memory of a late governor, and two others—books—were presented by the governors for proficiency in the medical and surgical nursing examinations. Besides these, the "Butterworth medals," given for five years' work in the service of the hospital, were presented to several sisters and nurses. Miss Nott-Bower welcomed her guests in an interesting little speech, in the course of which she mentioned various recent changes in the nursing department, notably the addition of thirty extra nurses, whereby the working day has been reduced from 10¼ hours to 8¾ hours. She also alluded to the establishment of the new recreation club, with its many privileges.

### THE CHILDREN'S FRESH-AIR MISSION.

ON Monday week the annual meeting of the Children's Fresh-air Mission will be held at the Mansion House, the Lord Mayor in the chair. Among the speakers on behalf of the fund will be the Bishop of Stepney, Sir Gainsford Bruce, Mr. E. Flower, M.P., Canon Newbolt, and Mr. Walter Hazell, M.P. It is now seventeen years since this excellent mission was started in a modest manner, and it is interesting to note that while in 1882 the managers were only able to send 269 children to the seaside or the country for a fortnight, last year upwards of 3,000 enjoyed this inestimable privilege; yet we observe that in 1895 and 1896 the number exceeded 3,300. In 1899 the record, we hope, may be passed. Very little assistance on the part of the public will suffice to ensure such an admirable result, for every gift of 10s. makes a holiday possible to one more child. It is not the least excellent feature of the mission that it is absolutely undenominational. Churchmen and Nonconformists join together for the sake of the little ones. The beneficent enterprise is confined almost entirely to the Holborn Union, and leaves general operations to the Country Holiday Fund.

### UP-TO-DATE GRATITUDE.

IT is never unprofitable to listen to the criticism of philanthropic schemes by those for whose benefit they have been devised, even though they sometimes turn benevolent intentions to ridicule. Gratitude is not infrequently conspicuous by its absence. Not long ago the wife of a labouring man studied in her parish magazine the rules relating to the employment of the district nurse. Finding that she was to work under the doctor, and was not expected to clean up the sick room, but merely to see that the work was done, the reader wrote in the most advanced nineteenth-century fashion to the local newspaper: "Of course, this step is taken to help the poor labouring class; but how can they benefit by it if they have three in attendance—the doctor, the amateur attendant, and parish nurse? I think that the labouring class of W—— must be well off to afford this." She concluded her comments by expatiating on her own difficulty in getting a nurse when she was ill, and by advocating the employment of a working woman to clean up. At the same time she condescendingly described the nurse in office as an "estimable young lady." To the poor the parish nurse is free; so frequently is the medical attendance. A labouring man's wife evidently would like the services of a charwoman into the bargain. In other words, she, who before the institution of district nursing must perforce have been content with the ministration of an ignorant neighbour, finds fault because the nurse does not relieve her of all the expense of her sickness.

### DISTRICT NURSING IN GLOUCESTER.

ON Thursday the annual meeting of the Gloucester District Nursing Society was held in the Guildhall, the Dean presiding. There was a good attendance, and the novelty of the proceeding was an address on the subject of district nursing by Mrs. Clare Goslett. The Chairman, in addressing the meeting, remarked that general approval was sometimes not so profitable as a little wholesome abuse. Everyone approved of this most needful and helpful institution, but at the present moment they were from £30 to £40 to the bad. It has been decided to buy a house for the use of the nurses. The hon. secretary's report also contained a strenuous appeal for a larger income. The committee deplore the loss, by death, of Dr. Ancrum, vice-president of the society since its institution, and of Miss Taynton, an energetic colleague.

### EMPLOYMENT FOR GENTLEWOMEN.

THE annual report of the Manchester Gentlewomen's Employment Association is full of instruction both as regards the work done and the difficulties which beset it. In the words of the Hon. Secretary its aim was "to help educated women, who were obliged to earn their own living, to do so in a satisfactory and suitable manner. The employment bureau, which had become only one department amongst others, was originally the nucleus of the entire association." The experience of such a society is invaluable, and the initial and greatest difficulty in carrying out the aims of the society is the lack of specified training amongst those for whom it was founded. So terrible, so hopeless was the fate of the legions of untrained women that the association raised a fund in 1893 for the sole purpose of paying the training fees of worthy educated women who are too poor to do so themselves. This number was found to be so great that the committee issued an appeal to the wealthier inhabitants of Manchester. The appeal has met with a generous response, and a grand bazaar will be held in November next, when it is hoped that £15,000 will be collected for the fund.

### DISTRICT NURSING AT BERWICK.

THE report of the Berwick Ladies' District Nursing Association was presented at the annual meeting recently. The Mayoress presided, and there was a good attendance. During the year the association has lost its first president, the late Lady Crossman. Her work and interest in the society was referred to in appreciative terms by the hon. secretary. Three Queen's nurses and twenty village nurses are now employed by the society in Northumberland, and its financial position is excellent. At the beginning of the year, in March, 1898, there was a balance of £45 in hand; the year closed with a credit account of £43, the income having been £240. In February the nurses' work was inspected by Miss Wade, who gave a satisfactory account of it and of the manner in which it was performed. Lady Frances Godolphin Osborne, of Orde House, who before her marriage helped to found the association, was elected president; and Lady Grey, of Fallowden, and Mrs. F. D. Blake, of Sanson Seal, vice-presidents. The Mayor, in proposing a vote of thanks to the retiring members of the executive committee, remarked that he had not at first regarded the scheme with favour, as he feared it might tend to demoralise the people, but since he had seen the excellent work accomplished by the association his opinion had completely changed.

### PSEUDO NURSING HOMES.

AN affecting little story was told to the magistrate at Bow Street the other day. A lady asked what she was to do with a little girl who had been left on her hands in infancy by a mother closely connected with the Imperial House of Austria. Considerable public sympathy was excited in the case, and the lady received several offers to adopt the child. Others made inquiries as to the advisability of sending contributions towards its support. But an enterprising reporter thought it worth while to investigate the lady's antecedents, which must have been the last thing she desired. About two years ago she was found to have sued a gentleman for the board and treatment of his daughter in a nursing establishment which she was running at the time. It came out in court, not only that she had neither nurses nor appliances for the prescribed treatment, but that the special diet was represented in her establishment by herrings and salt beef, condensed milk and cheap eggs! Such incidents not only emphasise the need for caution in giving promiscuously, but also prove the wisdom of using none but well-recommended nursing homes. As a matter of fact, swindlers often succeed in making money when bonâ-fide nurses come to grief.

### THE SHEFFIELD EXPERIMENTS.

THE proposed erection of the new nurses' home for Sheffield was challenged at a recent meeting of the Board of Guardians by Mr. H. Wells-Smith, because, he maintained, it was rendered unnecessary by the provision of aged people's homes, the extension of the scattered children's homes, the erection of a large test house for 300 people, and the recently adopted proposal to re-classify the indoor and outdoor paupers. Although the challenge produced no change in the scheme for building a thoroughly adequate and comfortable residence for the nurses, it is interesting from a nurse's point of view. It shows the enormous amount of work that falls to the share of the conscientious guardian, of which the care of the sick forms only a part; and it also proves that, however the policy of the Poor Law may develop, the work of, and provision for, the nurse must increase to keep pace with it. Sheffield, as many of our readers are aware, is the pioneer union, and the advance in our methods of dealing with social problems is largely due to experiments conducted by its Board of Guardians.

### SHANGHAI JUBILEE MEMORIAL.

A MEETING of subscribers to the Queen's Diamond Jubilee Memorial at Shanghai, which is to take the form of a nursing institute, was held early in the year to consider an important resolution. Mr. Wade Gardner presided, and proposed "that the committee should be authorised to accept contributions from donors of all nationalities; and that they should be requested to convey the thanks of the subscribers to the ladies who had offered to organise a bazaar in aid of the Building Fund." Since this resolution was passed some liberal contributions have been received from foreign well-wishers.

### BRAVERY HONOURED.

THE Royal Humane Society has just bestowed an award on Miss Ellen Hold for her bravery in jumping into the sea at Blackpool and saving the life of her patient who had accidentally fallen in. Nurse Hold performed this courageous act last February, and she and her patient were promptly charged after their rescue with attempting suicide! The story, with its appropriate conclusion, forms quite a basis for a romantic story. The accident to the patient, the attempted rescue by the nurse, the actual rescue by some fishermen, the charge of suicide by the authorities, and the award to the heroine by the Royal Humane Society could be turned to excellent account by a skilful novelist.

### SHORT ITEMS.

THE bazaar in aid of St. Mary's Hospital will take place at the Hotel Great Central, Marylebone Road, on June 6th and 7th. There is no doubt that the fact of it being held in a splendid suite of rooms at the newest of hotels will constitute an attraction; and it was politic, as well as generous, of the proprietors to offer the accommodation to the hospital authorities free of charge.—At a seminal meeting of the Sanitary Institute on Wednesday evening, Miss Alice Ravenhill opened a discussion on "Practical Hygiene Teaching in Elementary Schools." The chair was taken by the Rev. T. W. Sharpe, C.B., formerly chief inspector of schools. —Mr. and Mrs. Rider Haggard have taken much interest in the Ditchingham Nursing Association, recently formed to secure the services of a nurse for that and the neighbouring parishes of Hedenham, Broome, and Mettingham. The society has failed to secure a grant from the Local Board of Guardians on the score that it was unfair to the union to tax the whole for the benefit of a part.—A proposal has been adopted to inaugurate a regular three-year course of training for nurses at Keighley Workhouse. The stipend of the resident medical officer has been raised £25 on this account.—An additional permanent nurse has been appointed at Uttoxeter Workhouse.

### Typhoid Fever and Typhoid Nursing.

A Lecture to Probationers delivered by SISTER ELIZABETH, St. Bartholomew's Hospital.

TYPHOID or enteric fever is a disease in which the lymph follicles of the intestines become swollen and ulcerated, and in which there is swelling of the mesenteric glands and spleen, caused by the action of a specific bacillus. The parts of the intestine principally affected are Peyer's glands in the jejunum and ileum. The large intestine also is sometimes affected. The four stages of the intestinal lesions are: (1) Hyperplasia or swelling; (2) necrosis or sloughing; (3) ulceration; (4) cicatrisation or healing.

To pass on now to a general description of the disease, it may be roughly classified into four kinds, viz.: (1) The mild and abortive form; (2) the grave form, with high fever and acute nervous symptoms; (3) the latent or ambulatory form; (4) hæmorrhagic typhoid, which is very rare.

The incubation period lasts from eight to fourteen days, and is sometimes as long as twenty-three days. During this time the patient experiences chills, headache, nausea, loss of appetite, pains in the back and legs, and very often has attacks of epistaxis. At the end of this time the patient is fain to take to bed, and the first week of the disease is then entered upon, when the following symptoms are very generally constant: (1) A steady rise in temperature, characterised by a daily evening rise, and a morning remission; (2) a rapid, usually dicrotic, pulse; (3) a coated, white tongue; (4) headache; (5) slight distension and tenderness of the abdomen; (6) enlargement of the spleen; (7) cough and bronchitis; (8) appearance of rose-coloured spots fading on pressure, occurring in crops.

The second week of the disease is characterised in an ordinarily severe case by the following symptoms: (1) High fever without morning remissions; (2) a rapid pulse, no longer dicrotic; (3) mental torpor and dulness; (4) dry lips and tongue; (5) diarrhœa and tenderness in abdomen; (6) tympanites is increased.

In a mild, favourable case the temperature may decline about the fourteenth day, or in the reverse type, death may occur in the second week from hæmorrhage, perforation, or with pronounced nervous symptoms.

By the third week in moderately severe cases, the loss of flesh and weakness is pronounced, typhoid being essentially a disease in which extreme wasting and emaciation take place. The temperature now begins to come down with marked morning remissions. The pulse rate ranges from 110 to 130. The unfavourable symptoms at this stage are: (1) Pulmonary complications; (2) increasing weakness of the heart; (3) delirium with muscular tremors and subsultus tendinum. The special dangers to be looked for at this stage are perforation and hæmorrhage.

At the fourth week convalescence begins in the majority of cases, *i.e.*, (1) the temperature remains normal; (2) diarrhœa ceases; (3) the tongue cleans; (4) the appetite returns. But in severe cases the symptoms "may only be an aggravated picture of the third week" over again; the patient grows weaker, the pulse becomes more feeble and rapid, the tongue is dry, and the abdomen is distended. Profound stupor, with low muttering delirium, comes on. Jerky twitching movements, especially of the hands at the wrists, called subsultus tendinum, are to be noticed at this stage, and are usually of fatal import, though not always. Picking the bedclothes, and the involuntary passage of urine and fæces complete the gloomy list of bad symptoms to be met with at this stage in particularly severe cases. In such conditions death from heart failure and secondary complications is to be apprehended.

During the fifth and six weeks relapses of the disease may occur. The patient is also subject to irregular elevations of temperature, which, however, do not usually amount to much, and may be practically disregarded. At this stage the sequelæ of typhoid begin to show themselves, and thus convalescence is often much delayed.

It will be as well to enumerate concisely, before passing on to the nursing of typhoid fevers, the complications and sequelæ of the disease.

A. The principal complications to be met with are: (1) Bronchitis; (2) pleurisy; (3) pneumonia and hypostatic congestion and œdema of the lungs; (4) thrombi in the veins (in those of the left leg usually); (5) boils on the back; (6) hæmorrhage from the bowels; (7) peritonitis; (8) perforation; (9) parotitis; (10) infarcts and abscesses in the spleen.

B. The sequlæ of typhoid fever are: (1) Intense weakness, especially of the legs; (2) insanity and dementia, due, says Professor Osler, to "impaired nutrition and exhaustion of the nervous centres"; (3) periostitis, necrosis, and caries; (4) septicæmia and pyæmia, with multiple abscesses and boils.

The foregoing account will show you in some little degree the very serious nature of the disease you may be at any time called upon to nurse, and we may now pass on to some practical points of nursing in typhoid fever.

The bed should be a hair mattress on a wire frame if possible, accessible on each side. The bedclothes should be light and warm, and no vallance should be allowed. A water bed should be provided if the case turns out to be a protracted one, as the great wasting and the tendency to the formation of boils may lead, without great care on the nurse's part, to broken skin or even a bed-sore. The bed should be placed with the light coming from behind or from the side, and not facing the light.

WASHING AND SPONGING.—A patient with typhoid fever may be sponged all over night and morning as long as the temperature remains high, such a measure conducing much to comfort and well-being by reducing the fever, soothing the nervous system, and removing to a great extent the peculiar heavy smell so often met with in typical cases. Aromatic lotions are often mixed with the water, and are very agreeable to both patient and nurse.

The hair is much affected by the disease, inasmuch as it almost invariably falls out; so that it is much better to cut it quite short at the beginning of the attack. The head is then kept cool and clean, and the hair grows better afterwards.

The mouth must be regularly cleaned and washed out to prevent the lips from cracking, and to avoid the accumulation of sordes on the teeth and lips. In bad cases a nurse must do this constantly, and not less often than every two hours in any case where there is a tendency to foulness of the mouth.

The eyes in unconscious cases, where the patient does not close them, must be bathed regularly with boracic lotion to prevent ulceration of the cornea through dust, &c., settling on it.

The back must receive the nurse's special care and attention, as such patients are very liable to boils on the buttocks and sacrum, and troublesome bed-sores may result from them. Best of all preventive measures is to harden the skin with spirit lotions, of which brandy, whisky, or absolute alcohol are the best, and to keep the patient entirely off the back, by turning him from side to side every two or three hours, supporting the back and shoulders with a pillow. Should the skin break, or the top of a pimple come off, and threaten to be troublesome, a mixture of zinc oxide, boracic acid, and starch powder, equal parts, with a very little iodoform is most efficacious in drying up and healing such abrasions.

The position in bed is important for another reason besides the prevention of bed-sores. Hypostatic congestion of the lungs is a condition very likely to occur if the patient be allowed to lie on his back low down in the bed day after day; so, for this reason, the moving regularly from side to side and propping up of the back in this position should be religiously carried out by the nurse in charge.

FEEDING.—Liquid food only is advisable, and in most cases only permissible, till the temperature has been down for five or six days. Patients, while the fever is at its height, should be fed regularly at intervals of two or three hours with a stated quantity of milk or meat essence. The milk should be diluted with water, lime-water, barley-water, or whey, and should be boiled or peptonised besides when the digestion is much impaired. Boiled milk and strong extracts of meat are better strained before administration, to prevent any skin of milk or meat granules being swallowed. Beef-tea and meat essences are contra-indicated when diarrhœa is present. Water may be freely given to quench thirst. The quantity given should be from three to four pints for a woman, five to six for a man, in the twenty-four hours, and may be divided into 8 oz. to 10 oz. feeds at a time. During the night if the patient is soundly asleep it is as well not to wake him at the stated time, but to give more food when he does wake; but unconscious patients, or sleepless, restless cases, should be given food at regular intervals, as during the day-time. If there is sickness and diarrhœa, with undigested curd in the stools, whey and albumen water may be substituted for milk for a time. Stimulants may be freely mixed with the milk, whey, and albumen water. Tea, coffee, cocoa, barley water, milk jelly, buttermilk, and koumiss are all permissible, and help to vary an otherwise monotonous diet.

The following is a working model of a typhoid patient's daily diet in Elizabeth ward (women):—

| | |
|---|---|
| 7 a.m. | Coffee made with milk 8 fl. oz. |
| 9 a.m. | Milk (warm) ℥vi., water ℥ii. |
| 11 a.m. | Whey ℥iv., albumen water ℥iv. |
| 1 p.m. | Beef or mutton ess. ℥viii., strained. |
| 3 p.m. | Milk jelly ℥vi. |
| 5 p.m. | Milky tea ℥viii. |
| 7 p.m. | Beef or mutton ess. ℥viii. (as above). |

Custard and calf's-foot jelly is usually the first step towards a convalescent diet, closely followed by well boiled and beaten bread and milk, like bread sauce; next comes thin bread and butter without crust, boiled sole and pounded chicken and meat, till at last it is deemed safe to give ordinary diet.

THE EVACUATIONS.—The treatment of the evacuations is an all important one, and, as in all well regulated hospitals, the rules laid down here in regard to this point are duly stringent. I need only recapitulate to you the necessity for a thorough use of the prescribed disinfectant (1-20 carbolic) in the bedpan before and after the evacuation has been passed, the disinfection of the drains into which the evacuation is emptied, and the necessity for absolute cleanliness and spotlessness of the patient's bed linen, as the infection is conveyed by the urine and fæces, and more especially by the latter. After a nurse has attended to a typhoid patient she should immediately disinfect her hands and wrists, and wash them thoroughly with soap and water and a nail brush before doing anything else. Cases where there is constant diarrhœa are more dangerous to those in attendance than those where constipation is the rule, and in the former type of case, nurses on night duty should be specially careful, as the darkness makes it more difficult to avoid fæcal infection, and particularly in cases where liquid stools are passed involuntarily.

It has been difficult to compress an account of this formidable disease into the limits of a 45-minutes lecture, and in consequence style has had to be sacrificed to a utilitarian conciseness.

### The Matron's Corner.

### HIGH IDEALS.

A NURSE of long standing said to me once, "Oh, if only you could urge the importance of raising the tone of hospital life. A high ideal is so badly wanted." She had been many years in a big hospital. Since leaving it she has watched its working with critical but loving eyes, and she is sad because she sees a lowering of tone, a falling away from what I call "the great ideals of hospital life."

In our utilitarian age we have no lack of *practical* improvements everywhere, and hospitals go with the times in improving their general working to the minutest detail. I do not wish for a second to disparage these improvements, and, indeed, I shall propose, in later articles, that we should arrive at an interchange of opinions from matrons all over the country, as to every sort of change for the better that can be introduced into the working of a hospital. But just for the moment I want to step upon different—may I say, higher ground?—for we are so terribly prosaic nowadays that we are apt to forget the other side of the shield, the high ideal that should lie behind the visible life of every day. It is laying too great a burden of responsibility upon one pair of shoulders to say that it is the matron of a hospital who sets its tone, with whom rests the power of making it a place of great ideals, or of petty tyrannies and small aims.

It is the truth, nevertheless, whether they own to it or not, that the sisters of wards take their tone from the matron, the nurses and probationers take theirs from the sisters. It is, therefore, evident that to a conscientious woman the post of matron is no sinecure—it means so much more, or it *should* mean so infinitely more, than merely organising and ruling, important as are both these functions. But, you will ask, "What *are* these great ideals? It is our business, as matrons, to superintend the nursing in the hospital, to know that the sick are properly tended, the wards kept in good order, to see that the nursing staff is well trained, well fed, well cared for, that the servants and ward-maids do their work; that the housekeeping is well and economically carried out. We have a duty to perform to the hospital on one hand, to the committee and subscribers on the other." Yes, all that is your business, and more; you have to imbue those working under you, by your influence and teaching, with the highest aim of nurses, *e.g.*, to be the "ministering angels" of life. You have to show them—not by absurd and stringent rules, broken behind your back—but by noble example and loving words, that a hospital is not a place for folly and frivolity, but rather one where each nurse may learn to be,

> "To other souls
> The cup of strength in some great agony."

You should make your nurses see that their time of training is a time for strengthening and ennobling their own characters; and that in learning to nurse the sick they ought also to learn pity, and love, and sympathy for humanity in its every shape and form. You must imbue them with the noble spirit of the service of humanity. It is the matron who should keep these ideals before the eyes of her subordinates. From her presence and her life they can learn also to "be the sweet presence of a good diffused." I have hinted at some of the great ideals to be aimed at by our hospitals, and I should welcome discussion from any of those who are engaged in the difficult work of hospital government. I wonder if it strikes them, as it does me, how suitable these words would be above our matrons' offices, over the doors of our sisters' and nurses' rooms: "Strengthen ye the weak hands, and confirm the feeble knees; say to them that are of a fearful heart, Be strong, fear not."

### Post Graduate Clinics for Nurses.

### THE USES OF WATER IN THE SICK ROOM.

### II.—Hot and Cold Water, Dripping Sheet, &c.

Considering the bath as a form of treatment I will briefly mention the chief points aimed at in hot, warm, and cold baths and packs. The hot bath is given at a temperature of 98 to 112 deg. Fahr., and is either local or general according to the effect it is desired to produce. The first principle involved in the application of both hot and cold water is that either of these produces a marked reaction. A warm bath is not followed by reaction, consequently it is not to be compared from the point of view of treatment with either hot or cold water. We are all familiar with the fact that hot water —either in the form of a bath or of a fomentation—produces distension of the arteries and increases the vital activity and temperature of the part to which it is applied. Prolonged hot fomentation causes the blood vessels to relax more or less permanently and so to relieve congestions. It is for this reason that prolonged hot sitz baths are given to relieve pelvic congestions, &c. The hot bath artificially raises the bodily heat proportionately to the temperature of the water, and much increases the rate of the pulse. Because of this stimulating and excitant effect, hot baths do not suit all patients, and the nurse should carefully watch for symptoms of flushing and faintness in cases for which hot baths are ordered. The hot foot-bath has sometimes the same effect of causing dizziness and faintness through over-stimulation. In many excitable patients the hot bath produces sleeplessness, and this result should of course be reported promptly to the doctor, who may in such a case order the hot bath some hours before the usual sleeping time, so that the excitant effect of the bath will have worn off before the sleep hour, and thus will not interfere with a good night's rest. In some other patients, in whom hot bathing produces a feeling of faintness and exhaustion, the bath seems to act as a sleep-producer. The opposite effects on differing temperaments should not be lost sight of by the nurse. Nurses must remember that a bath at 100 deg. and upwards may in a very short time increase the patient's pulse from the normal to the rate of 102 to 120 beats per minute, which explains why a patient under hot-bath treatment needs watching. If a feeling of faintness occur while the patient is in the bath a cold, wet towel should be applied to the head, cold water added to the bath, and the patient lowered as nearly horizontal as possible. When the hot bath is used to excite and promote the action of the skin, the best plan to adopt is to begin the bath at 99 deg. or 100 deg. Fahr., and to raise it by the gradual addition of hot water to a temperature of 108 deg. to 110 deg., allowing it to remain at the latter temperature for about ten minutes. After this the patient is wrapped in blankets and thoroughly sweated for two to three hours. The gradual raising of the temperature lessens the excitant and stimulating effect on the heart and blood vessels, and allows the bath to be taken at a higher temperature. Few patients could bear to be suddenly put into a bath at 108 deg., but gradually heated no inconvenience is felt. This form of hot bath is much employed in kidney disease, where it is important to obtain a free action of the skin. The warm bath is used more as a cleansing agent than for purposes of treatment, although it is very commonly prescribed as the forerunner of cold water treatment—that is to say, a patient whose temperature needs reduction is put into a warm bath, and this is gradually cooled down, or else as he sits or stands in warm water pailfuls of cold or colder water are dashed over him.

Warm baths differ from cold in that they produce no shock and are not followed by reaction. At the same time immersion in warm water has a great power of causing skin activity, and the warm bath—and by this is meant a bath from 85 to 98 deg.—is soothing and hypnotic.

Warm water is employed in the perpetual baths of Germany and Austria, wherein the patients remain under water for many months and in some cases upwards of a year, while at several Swiss bathing-places patients under treatment spend the whole of each day in the water provided with floating tables, on which are placed their meals, books, and games. The warm bath is most soothing in cases of nervous irritability.

Turning to the cold bath, there is very little to say from the point of view of the sick person on the custom of cold bathing. Cold or cool water applications and affusions are, however, so general in the treatment of fevers that the various methods adopted to bring down temperature will be described in turn.

A cold bath ranges in temperature from 33 deg. to 60 deg., and it has an entirely different effect when prolonged from what it has when given as a plunge. A quick plunge is accompanied by some shock and followed by reaction, while a prolonged cold bath is sedative. The brief cold bath temporarily contracts the blood vessels, to be followed by reactive dilatation and generally increased blood supply. A cold bath maintained causes the blood vessels to remain contracted for a long time—hence the value of cold water in bringing down temperature. A cold bath prolonged may decrease the heart beats as much as 20 to 40 beats per minute, and may lower the blood temperature from one-half to five or six degrees. So that cold water differently applied has opposite effects—stimulation on the one hand or a remarkable power of causing vital depression. Cold water treatment is much used in the modern treatment of the continued fevers, typhoid, and hysteria, while the wet brief pack proves very useful in mania, delirium, sunstroke, and many other conditions. Undoubtedly such treatment would be much more common in practice were it not that the application of water in any of its myriad forms is somewhat troublesome and calls for experienced and trained assistance. The "dripping sheet" is very useful in hysteria and melancholia, or for bringing down temperature, and is easily given. The patient stands in a bath in a few inches of water at about 100 deg., and the nurse dipping a sheet in water at about 68 deg., wraps it dripping round the patient, vigorously rubs the entire body up and down, dashing water 10 to 15 deg. lower than that used for the sheet all over the patient. A brisk rub down afterwards completes this restful and fever-reducing bath. When this bath is given to bring down temperature it should last at least fifteen minutes. If the sponge bath is given to reduce the fever of a patient in bed it is best to use water at about 98 deg. for the first sponging, and to gradually reduce the temperature of the water by adding ice and methylated spirit till the last sponging is with water at about 45 deg. After bed-bathing for the reduction of temperature it is not necessary to dry the patient limb by limb as in the ordinary cleansing bath. After the bath he can be wrapped entirely in a sheet, dried thoroughly, and chill guarded against.

### To Nurses.

In order to increase and vary the interest in the *Mirror*, we invite contributions from any of our readers in the form of either a paragraph, a letter, or information, and will pay a minimum of 5s. for each contribution.

### Across the Seas.

### SOUTH AFRICAN HOSPITALS.

### II.—Kaffir Help.

In my last paper I pointed out that the endeavour to train the colonial nurse upon the English pattern has resulted in her losing caste both in Kaffir and colonial estimation owing to her being put to the duties of the broom and the brush. While the Kaffir dimly appreciates that the performance of such domestic work by a religious sister is in some mysterious way a penance belonging to her cloth, when the same work is done by a lay nurse he dubs her "white nigger," and feels that he is on a perfect social level with her. In fact, unless particularly low-class Kaffirs are appointed as ward-maids and helpers, they refuse to perform "menial" and objectionable work. And where the "English method" is in vogue and the nurses are expected to perform the heavier of the domestic duties, the work which black Kaffirs refuse on account of its hard and unpleasant nature is very apt to fall to the lot of the youngest and meekest probationer. The pro, being a colonial, resents more than an English girl having to do "Kaffir leavings," and in the bitterness of her heart is apt to wax eloquent on the subject of customs imported and transplanted from England, where they doubtless work well, to South Africa, where they have undoubtedly not proved a success.

Another very serious difficulty has been introduced into South African hospitals by the establishment of the "English principle" that male patients should be nursed by women nurses. No doubt this is an admirable working principle for civilised England, where the average man, however degraded, has a perception and instinct of respect for a refined gentlewoman. Here the system of putting a cultivated woman in sole charge of a Kaffir ward, and making her perform all the necessary duties towards such patients, has proved so revolting in its working that a strong prejudice has arisen in the minds of educated colonial parents against allowing their daughters to train as nurses. It would be impossible for me to convey even a fragmentary impression of the habits and customs practised in a Kaffir ward. The language and the unspeakable conduct of the average male Kaffir in hospital are such that the nursing of these patients would prove a trial to a superior native woman. Yet a Colonial or English girl of 23 is oftentimes in charge of such a ward, and at night is on duty alone amongst these primitive and degraded creatures without the assistance or protection of an orderly, a watchman, or a porter.

A romantic sentiment as to the "poor native" is all very well—as a sentiment. But you do not hear much of that kind of romance from a person who knows the Kaffir. The Matabele is a different person. His type is much higher in the human scale, and he is kept under stringent control se that he cannot obtain alcohol. Yet despite Matabele superiority no woman nurse in the Rhodesian hospitals is put in charge of a native ward. The work is done by white orderlies assisted by native helpers, the whole being super intended by white sisters or nurses.

In too many hospitals of Cape Colony the entire nursing of the native Malays, Kaffirs, half-breeds, &c., is confined entirely to colonial or English women nurses, but we are hoping to banish this among our imported nursing fashions. The mixture of the black and white races—productive of myriads of half-breeds—is a problem which in the future will have to be met in the South African training schools. So far few half-bred nurses have applied for training in our hospitals, but this development will have to come, and the difficulties which it will involve can only be understood by those who understand South Africa and the habits and customs of the "black and white mixed."

To sort out the different shades of tan is a very onerous task, and however much the matron may wish to keep the coloured races from her training school some of them creep in as creoles. Indeed, if you were to believe the statements of the coloured women of South Africa you would come to the conclusion that none of them were born in the colony at all. They all claim to hail from St. Helena and Mauritius.

Lest my objection to the training of coloured women as hospital nurses may appear narrow-minded, I should like to point out that this is a subject which the English person who has never been here cannot judge. While I am extremely hopeful that the time will come when it will be possible to train the Kaffir woman as a hospital nurse, I must frankly confess that such a millennium is far off. Eventually, of course, the half-breeds will shake down to their due social standing, but at the present time the low standard of education and morality of the mixed race makes their introduction to the privileges of a hospital training school undesirable. The pure Malay or Kaffir is out of the question for lack of knowledge. As a ward-maid and hospital cleaner she proves a somewhat difficult person to manage. It is futile to hope for a time when our hospitals will be staffed entirely with white people, since the coloured races are increasing so rapidly. Tuition and training will accomplish much, but the Kaffir in a South African hospital is one of the trials which the conventional English nurse "just out" from home finds the hardest to bear. The colonial woman has been born and bred to Kaffir ways, and understands better how to deal with them and their subterfuges.

But the sufferings of a nurse trained in London who subsequently finds herself "in charge" in a South African ward, with its complement of queer Kaffir help, are very real. And though the courageous Englishwoman will fight down sooner or later the domestic difficulties occasioned by native workers, without plenty of resolution to face such trials she is not suited to pioneer colonial life.

At first the English matron, confronted by a household of Malays, Kaffirs, and half-breeds, feels that the situation is too much for her. But if she be a sensible, practical woman —and these are the women we want here—she makes the best of the bad domestic bargain and works away to bring order out of chaos. The most difficult of all the native habits is their irresponsibility and the happy-go-lucky temperament which the least touch of the tar-brush appears to engender. The absolute and almost ludicrous want of conscience as to the performance of allotted duty, which seems inherent in the South African native, adds considerably to the complexities of life in a colonial hospital. It "isn't their fault if the tea is made with cold water," the fault rests with the person who took the boiling water just as they were going to make the tea, &c.

The sister must always be prepared for the ward-maid to take a day off without permission, or to come on duty three-quarters of an hour after the due time. It seems impossible to imbue the average Kaffir with any sense of punctuality or discipline. On Saturday nights the ward-maids and Kaffir maid servants go to dancing saloons and stay until the early hours of the Sabbath. They make their appearance at an unconscionably late hour on the Sunday morning, and inform the matron, sisters, nurses, and all others in authority that they "don't approve of Sunday labour, which is against Christianity." This latter tenet of the Church is the only one they care to hold fast to. For though many of them profess to be "converted," the profession is often made with the object of getting education and clothes, and they still continue to patronise their own "spooks" and witch doctors.

### Echoes from the Outside World.

AN OPEN LETTER TO A HOSPITAL NURSE.

THERE was never a woman yet—unless, perhaps, the most hopelessly blighted old maid—who did not take an interest in weddings, and though, except at holiday times, nurses seem to have more to do with the sad than the sunshiny side of life, I know you dearly like to hear any odd scraps about Love, Courtship, and Matrimony. Not because you look upon it as the only thing in life—that degrading idea of living only to try and get married has, I am proud to think, been destroyed by the good, honest, helpful work most women take up now-a-days—but if a Mr. Right ever happens to come along and claim one of our friends, or even someone whom we only know by reputation, of course we like to know all about it. Have you heard the rumour, which returns so persistently again and again that at last it seems as if it must have a foundation in truth, that the engagement of Princess Victoria of Wales to her cousin, Prince George of Greece, will shortly be announced? The young people are said to have been in love with each other for some years, but like many another man in a humbler sphere of life, the prince felt that he had not enough to offer to justify him in getting engaged. Since the Governorship of Crete has been accepted by him his position is no longer unimportant, so those who ought to know say that during the coming season the news will be given to the world.

---

FOR the present the wedding of the Earl of Crewe and Lady Peggy Primrose on Thursday is occupying most attention. Primroses are likely to be more in demand than ever this year, for the 19th is the day devoted to the memory of Lord Beaconsfield, when the pale yellow blossoms are always in great request. At the ceremony next day thousands of bunches will be employed in decorations and otherwise, for I am told the marriage is in its way to be quite a Primrose demonstration. The bride, who, you know, is only eighteen, ought to look very sweet in her bridal gown, and many are the good wishes for her happiness, though of course there are not wanting people who shake their heads about the disparity of age. But really, if Lord Rosebery has no objection to the match, I call it impertinence for the general public to object, don't you? I have not yet heard what Lady Peggy's gown will be, but many of the April brides—and their name is legion—are going to wear lace, and even muslin over silk or satin. In fact, everything points to a "lace summer" as far as dress is concerned.

---

ALTHOUGH the Princess of Wales has done little to favour the movement (having worn the deepest crape for her mother), there is an increasing feeling among the middle classes that unless for very near relatives the wearing of heavy mourning is a mistake. For years and years different societies have been trying to bring about this reformation by binding their members not to go into black for relations, or at most only to wear a black band round their arm or a little badge of some description indicating their loss. But no real advance seems hitherto to have been made, though, I suppose, all efforts have unconsciously tended in the same direction, and now at length the result of past endeavours is becoming apparent. I heard of a family the other day who had sustained the loss of an aunt. The lady had lived some distance away, and the two families were not likely to meet. The question of mourning was discussed at the breakfast table, after the letter announcing the death had been opened. The conversation was naturally mostly confined to the ladies, but suddenly the father, a quiet, thoughtful man, made a startling remark: "If you girls like to make up your minds not to go into mourning I will give you a five-pound note each. If you do go into mourning I won't give you a farthing beyond your ordinary dress allowance." There is no need to add which course the daughters pursued.

---

Do you take any interest in the wireless telegraphy of which the papers just now are so full? I did not, because I somehow ran away with the idea that the subject was far too difficult for an ordinary brain to grasp. But I was calling the other day on a newly married girl, and she started the topic in such a matter-of-fact way as if every intelligent woman was following the subject closely, that I felt ashamed of myself and persuaded her to enlighten me. (She confessed afterwards that her husband had carefully explained it all to her, or she would have known no more than I did.) I feel rather diffident in trying to repeat her explanations to you as they will naturally be most unscientific, but it appears that by means of a very strong electric current, generated by powerful machines, the message is passed along a wire running up a tall pole. From the top of this pole the electric current travels into space. But a similar pole placed at a distance attracts these wandering waves of electricity, the wire passing the message down till it reaches the receiving machine below. I am told that the longer the distance to be crossed the higher the pole must be. The pole which successfully transmitted a message from the South Foreland to the other side of the Channel to a place close to Boulogne, was 150 ft. high, and though the experiments were tried during the gale of Friday, when the wind was blowing fiercely and the rain driving apace, there was not the slightest difficulty in reading the words. The use of this system, which is the marvellous invention of an Italian, Signor Marconi, is shortly to be tested on board ship. One of the cross Channel steamers is to have a receiver placed on board, and it is believed that as soon as the electric influence from the top of the pole on land comes into a line with the machine on the vessel, a little bell will ring warning the crew of the fact. It is easy to see the benefit of this should the test prove satisfactory, for given a pole with the needful apparatus erected near the Casquet lighthouse, and a receiving instrument on the "Stella" the captain would have known directly he came into a line with the electric current, and steered away again from the fatal rocks. Such at least are the hopes entertained, for fog has no power to stop these electric messages. Installations are to be set up in many places, and there is even some talk of communication being established at an early date between London and New York.

---

IT is a pretty little tale that comes from Berlin, and sounds almost like a leaf from Hans Andersen's Fairy Book. The poor woman in her humble abode with her triplet of little boys, happy in the possession of her darlings, but borne down with anxiety as to how to provide for three babies, two having come so unexpectedly. Then the sudden arrival of numerous bundles containing baby clothes, which all appeared to have been brought by magic hands, followed in a few days by an Imperial carriage containing two ladies and a gentleman. The occupants ordered bread to be sent up, took bags of cakes in their hands, and ascended to the fifth story. Here one of the ladies admired the babies, praised the cleanliness of the room, promised a perambulator which should also act as a sleeping couch for the infants, gave the astonished mother some money, and hoping the little ones would grow up "good men," left the room. The other lady stopped behind one moment to whisper the fairy godmother's name. It was the German Empress!

### The Ethics of Nursing.

EQUABLENESS.

Suppose that most people will agree with me that few qualities are more essential to a good nurse than that rare and delightful possession—an equable temperament. It is difficult to over-estimate the benefit to patients of being tended by a person of even temper and well-balanced mind. It is equally hard to exaggerate the ill effect produced upon invalids by the kind of person I have heard described as an "up and down woman." The words exactly fit the case. Those women who lack balance—whose temperaments are the reverse of equable—are so very much "up and down people." You literally "never know where to have them." One day they are in wild, overflowing spirits, full of laughter, talk, gaiety; the next, to your surprise, you find them downcast, sad, and silent; and all apparently for no reason. There is, as a rule, no reason, except that they will tell you they can't help it; they have the blues; they have the "hump." They go about with dejected faces, and if they are nurses they not only are depressed themselves, but they succeed only too admirably in depressing their patients also. I would say emphatically "to those about to become nurses," "Cultivate an equable temperament as one of your preliminary steps." The sick folk under your care will look to you for something more than the tending which you will, of course, give them ungrudgingly. They will want brightness from you, and freshness, and strength. Because their own minds and bodies are unstrung and unbalanced through illness, they need from those about them everything that is most strong and balanced and healthy. Now, to my mind, there is something distinctly unhealthy in one of those up and down natures, which are, so to speak, "all over the place"; and this is most trying to invalids, whether in a hospital ward or in a private house. It is very upsetting never to be sure what to expect from your nurse. It is bad for a patient, both mentally and morally, and, therefore, physically as well, to watch the door anxiously with the thought, "What mood will nurse be in to-day? Lively or doleful? Good-tempered or snappish In the heights or in the depths?"

Nothing will ever persuade me that an equable temperament cannot be cultivated. True, some fortunate people are endowed by nature with this most precious gift. But for those who are not its lucky possessors I say, "Cultivate it." I shrewdly suspect that a habit of unselfishness is a primary help, a fixed determination to think first what will make others happy, to put ourselves in their places, and to see with their eyes. For instance, put yourself in the place of your patient, and think what it would feel like to have a gloomy and depressed being hovering over you one day, a lively and talkative one the next, a bustling, irritated, worrying one on the third. Then you will have a dim idea of the effect upon the patient of a nurse of an unequable temperament.

A strenuous fight against a habit of introspection is another aid to equableness, for nothing tends more to general unhealthiness of mind, with depression and blues in its train, than that peculiarly foolish and tiresome custom of digging up one's soul to see how it is getting along—that perpetual gaping into the windows of one's own mind, instead of looking out into a sunny and workaday world. Introspection is a snare of the evil one, and I defy anybody to cultivate an equable temperament or any other virtue who also indulges in that bad habit.

"Look up, not down; look out, not in" is a grand motto. Let your patients be able to say, "She is always the same." Do not be either fussily hurried or wearisomely slow, either over-chatty or depressingly glum—cultivate the golden mean of equableness. The brightness that is not boisterous, the sympathy that is never emotional, the gentleness that is not over-softness, the strength that never degenerates into hardness, these will help you to be "always the same."

I heard it said once of somebody (she was a nurse then, by the way)—"You are just like a seabreeze." The speaker meant that she was refreshing and bright, and it was to that same person that a fellow nurse said one day, "Oh, how I wish I had your equable temperament." That lucky person had Nature's royal gift; the rest of us must attain to it by stress and struggle. But, believe me, it is attainable.

"The sunshine came along with him." Do you remember those words of our great poet? And can we not picture the passing of that sunny-faced youth bringing happiness and brightness in his train? Shall not each of us also bring, as we pass along life's ways, "the sunshine" with us?

### The Nurses' Bookshelf.

The Englishwoman's Year Book, 1899. (London: Adam and Charles Black. Price 2s. 6d.)

The "Englishwoman's Year Book" has been published annually under the guidance of Miss Louisa Hubbard since 1881. It has always been a useful little volume. This year it appears in a new form, greatly enlarged in scope, and the various branches of work in which women are occupied have been carefully classified. On turning over the pages of this good-sized volume one is struck by the thoroughness with which the task has been executed by the editoress and her colleagues, and by the important part which women now play in the work of life in England. Apart from the space devoted to employments there is the most complete information on all subjects affecting the position of women. Institutions for their benefit, chapters on housekeeping and kindred subjects, postal information, lists of books, papers, and magazines written for women, notes, and in short, all the knowledge collected to make the "Englishwoman's Year Book" as comprehensive as its title suggests. Miss Janes, who is secretary of the National Union of Women Workers of Great Britain and Ireland, is admirably fitted to continue the work of editoress so ably carried on until now by Miss Hubbard. She has a task worthy of her gifts in the compilation of a book which will not only prove useful to those who have already found their place in the working world, but which will serve to inspire many who through necessity or choice desire to find an opening for the exercise of their energies and abilities; for the book before us demonstrates that there is work to be done suitable for women of every condition and capacity. The editoress is in earnest in wishing to render her work as complete and correct as possible, and in order to further her desire, blank pages are inserted in the book for the notification of errors or omissions. This should be useful, as no compiler can hope to attain complete accuracy, however careful. But we fail to realise the suitability of inserting a page for a record of "family events." The "Englishwoman's Year Book," to fulfil its intention, should be placed ready for the reference of all and sundry, and therefore is hardly the place to insert a comparatively private diary. This is a criticism which in no way affects the excellence of the work, which we trust will soon be as widely known as it deserves.

### Guy's Hospital.

On Monday, March 27th, Dorcas Ward was re-opened, and the general move of the Women's Surgical Wards commenced, nine new probationers entering to facilitate the work. Miss Godby (Sister Charity), moved into Dorcas; Miss Fox (Sister Martha), into Charity; Miss Davidson (Sister Lydia), into Martha; and Miss Crawley (Sister Eva, medical night sister), took up her duties as sister of Lydia Ward. In each case the head nurses and probationers moved with the sisters.

### A Book and its Story.

"PROFESSOR HIERONIMUS."

THE interest of this remarkable and powerful piece of work* is enhanced by the fact that the authoress has herself gone through the scenes she describes with such painful minuteness. Fru Amalie Skram, the chief events of whose life are given in an interesting preface, is a Danish writer, a novelist of the realistic school. The circumstances which gave her the idea of writing "Professor Hieronimus" are thus stated in the preface: "Seeking quiet and treatment for a nervous affection, Fru Skram of her own free will became an inmate of a lunatic asylum. Thus she had a chance of studying one of those specialists in mental disease who are too apt to mistake rebelliousness for a sign of mental derangement." The story told in the book is thus practically her own experiences, the chief difference being that in the novel we have Fru Else Kant, the painter, instead of Fru Amalie Kram the writer. Else has been reduced by overwork, by her inability to express on canvas the ideals in her mind, by anxiety as to her child's health, and by consequent insomnia, to the point of attempting suicide. Once before insomnia had so affected her brain that she had seen hallucinations; but a stay of a month or two in a retreat kept by a kindly doctor, now dead, had restored her to her normal health. Therefore in her present condition she accedes willingly to her husband's suggestion that she should go for a short time into the hospital kept by Professor Hieronimus, a doctor who, as she says afterwards, when she has had some personal experience of his methods, was "a man who had published a few articles which had made a sensation, and who gave lectures that interested the young medical students. Nothing else was known of or about him. Yes, it was also known that he had a weak digestion, and a somewhat peevish temper."

The theory of Professor Hieronimus was that every one who was brought to his asylum was mad. But, even so, it was strange to put a woman suffering, as Else did, from insomnia, into a ward of excitable lunatics, where silence never obtained day or night. To make sleep even more impossible the ward below was occupied by maniacs, whose cries reached the corridor above. The doors of the various cells were never closed, so that when one patient dosed off to sleep she was liable to be awakened by some wilder or more restless neighbour rushing in and rousing her. Else's companions in misery are well described. A puerperal maniac is tormented by the thought of her children. She had tried to kill her baby because her husband was poor and they had so many children already. A flighty woman, who had gone mad with vanity, insists on confiding to Else stories of her admirers, her husband's jealousy, and her infidelity to him. A patient suffering from delirium tremens is placed in the cell next to hers; another lunatic manages to commit suicide. It is obvious that a nervous woman, whether mad or sane, could not have been worse housed than Else was in Professor Hieronimus's hospital. Her sufferings are narrated in full detail, from her being forcibly undressed when she enters the hospital, a bottle of cough mixture she had brought with her taken from her for fear that it was a poison, her garters removed, and the ribbon with which she bound her hair cut in half, lest she should use either to take away her life. The ridiculousness of the precautions, as applied to a sane woman, the bewilderment with which Else at first regards them, and the indignation she feels when she realises their intention, are well depicted. The various nurses with whom she comes in contact are discriminated with care. They are all kindly women, but all in terrible fear of the Professor, who holds them responsible and scolds them severely when a patient complains of the conditions of asylum life.

The portrait of Hieronimus is certainly drawn with a pencil steeped in gall. Not only does he start with the assumption that all his patients are mad when they come to him, but he treats them with a lack of tact, of decent common sense, which one does not expect in a specialist on mental diseases. He forbids Else's husband coming to see her, and even wrings her heart by concealing from her how anxious he is to do so. Yet he thinks to do her good by inviting to visit her a man whom she knew only slightly, as a society acquaintance, not realising that to a sensitive woman it is humiliating to be introduced to anyone as an inmate of a madhouse cell. The picture of Hieronimus is not without touches of grim humour. The doctor, who has no sympathy with his patient when she complains of the continual noises, starts nervously when a door bangs, and gives irritable orders to have the sound prevented. After he has come to the point of frankly telling her, as he has already told her husband, that she is mad, he goes on to explain that he has been helped to come to that conclusion by studying her pictures, which show, he says, such an interest in the abnormal in life as to indicate a strain of madness in her nature. Else wonders, though she does not dare to express her wonder, how one should regard that element in the Professor's nature which made him take up the study of the abnormal in humanity. And, indeed, all through the book we are made to feel that the attitude of Hieronimus towards his patients is a form of insanity on his part. He is, at least, a man of narrow intellect, and is utterly lacking in sympathy, which is an essential part of the equipment of any doctor, and most of all in one who undertakes to "minister to a mind diseased." He is deceitful, too, promising things which he has no intention of performing; withholding information from both husband and wife; opening the letters the former writes to the latter; doing and leaving undone as suits his imperial will. It is said that in Denmark the book had a great success as being drawn from life, and that the doctor for whom Hieronimus stands was at once recognised from the portrait given by Fru Skram. Here, of course, that doubtful interest does not apply to the book; it must stand on its merits. From an intellectual point of view these are undeniable. The book is as powerful as it is painful. It also suggests questions as to the treatment of patients in asylums, and the risk of the natural irritation of a sane person at being regarded as a mad one being looked upon by a prejudiced observer as a proof of insanity. Still, the present critic, having known one or two asylums, does not think that a prototype of Hieronimus would be easy to find in this country. Yet the story makes one realise the importance and seriousness of the task confided to the superintendent of an asylum, and the importance of choosing a man both shrewd and sympathetic. A word of praise must be given to the admirable quality of the translation. It has the supreme merit of not seeming to be a translation at all.

* "Professor Hieronimus." A Novel. Translated from the Danish of Amalie Skram, by Alice Stronach and G. B. Jacobi. (John Lane: The Bodley Head, London and New York. 1899. Price 6s.)

### Presentations.

MISS ELLEN TAYLOR, who has been one of the assistant matrons at the Three Counties Asylum for the last six years, and who has recently been appointed Chief Nurse to the Birmingham Asylum, was last week presented with a beautiful clock subscribed for by the nurses, and also a marmalade dish from the female patients. Miss Taylor's well-merited promotion has caused general satisfaction among the officials and inmates of the asylum, her genial and straightforward manner having gained for her affection from all with whom she came in contact.

### Spring Novelties.

### SPRING MATERIALS.

BEAUTY of colouring and design are the leading features of the spring novelties from Messrs. Egerton Burnett and Co., the famous Wellington firm. The choice is so varied and extensive, it is a little difficult to know where to begin, and whether the ornamental or the useful should take the precedence. A thing of beauty is a joy for ever, whether we require it or not, so we will commence with a brief description of the former. Delaine, that graceful material which lends itself so readily to the dressmaker's art, is re-asserting itself, and very beautiful are some of the designs we have before us; flowered muslins in a variety of shades are also another line of great attraction. Charming in appearance are the zephyrs, which have the effect of silk in the softest hues. One in blue and another in soft dove colour would make ideal costumes. It is with a considerable feels ing of pleasure that we welcome the reappearance of an old friend—Japanese silk, in a lovely shade of grey. Picture a costume of it trimmed with soft black lace, could anything be more becoming? Endless are the designs in washing material; drills being among the foremost in importance, and to our readers probably the most useful. Nurses will be difficult to please if they cannot find something out of the number that would fulfil their highest aspirations. Cloaks are made from any design on receipt of measurements. Patterns of the cloths will be submitted in each case with list of prices. Tailor-made skirts and costumes are made to order at any time, and if required a whole outfit can be provided at a most moderate cost. Serges, which are the great specialité of this firm, are more extesnive in their range than ever, and may be had in the finest to the heaviest quality. White flannels, washing serges, and Scotch winseys are also offered in endless variety, and anticipate the summer days that before long we have every reason to hope will smile upon us. To be forewarned is to be forearmed, and the advice we give our readers is to begin at once like prudent managers and set their wardrobe in order with the aid of the really charming materials that will be forwarded post free on receipt of a post-card.

### WARD SHOES.

"EASE before elegance" is the not unfrequent exclamation of the weary probationer as she regards her swollen feet in their possibly comfortable though inelegant coverings. H. E. Randall (Limited) are to be congratulated in having brought out shoes which are not only comfortable, but are both neat and becoming. They are very soft and pliable, readily adapting themselves to the shape of their wearers' feet, and with just sufficient heel to give support to the instep. The indiarubber with which the heels are capped renders them noiseless, and therefore these shoes are eminently suitable for night wear. The price is only 4s. 11d., and from the appearance we should be inclined to prophesy a quick sale and a constant demand. They are firmly and strongly made, and the sole projects all round beyond the upper leather, thus affording a firm tread. Those of us who have experienced the difficulty of procuring a really comfortable shoe will have reason to be grateful to Messrs. H. E. Randall. Branch establishments will be found at 366, Oxford Street; 68, Piccadilly; 134, Cheapside; and 14, High Street, Kensington.

### FOR NURSES' APRONS.

MESSRS. GARROULD, of Edgware Road, especially lay themselves out to meet the wants of nurses. In the choice of apron materials they know that nurses' wants are varied. With this in view they have secured the most extensive range of linens of all qualities, especially manufactured for them at Belfast. The Tikord Apron Linen, as it is called, is made in three widths, so as to minimise waste. A most serviceable linen is to be had as low as 1s. 4½d. a yard, 54 inches wide; and one of 45 inches in width costs less than this. The finest qualities are also to be had, and all are moderate in price.

### A SENSIBLE CORSET.

WE can confidently recommend to our readers' notice "The Twentieth Century Corset," which has been submitted for our opinion. It combines all the attributes which should be found in a perfect corset. The shape is good, it is light, and being made of firm strong net, it is well ventilated. The sides are laced with good elastic and allow for lung expansion without destroying the essential supporting properties. The corset is likely to find favour with all, but for invalids who could not endure a less comfortable support, for nurses, and all those engaged in active occupations the corset will be regarded as essential when once adopted. Either grey or white, with long or short busks, can be procured. Those with short busks are especially designed for cyclists and for riding. There is only one quality and one price, namely, 10s. 6d. The corsets can be ordered at any draper's or from Dr. Lahmann's Underclothing Agency, 15, Fore Street, London, E.C.

### Invalid Furniture.

### MESSRS. FARMER, LANE, AND CO.

IN the world of invalid furniture and appliances, Messrs. Farmer, Lane, and Co. hold a high position, and if ever a firm deserved success it may truly be said that they have

CHAIRS AND BED REST.

done so. Ever ready to take advice, always anticipating their customers, needs, it is not surprising that their reputation has increased by leaps and bounds. The workmanship exhibited in all their goods is of the very best possible kind; there is nothing shoddy or second-rate on the premises. Though not exactly cheap, because cheapness is incompatible with the best class of work, their prices are most reasonable, and no purchaser has ever had reason to regret his dealings with the firm. Couches and every description of chair are the most notable of the many excellent articles that a visit to 77, New Oxford Street, reveals. Among the former is a delightful contrivance in three sections, which can be adjusted at any angle to suit the position of the body, and a reading-stand attached that swings round as required. The inexpensive Ilkley couch is another variety, not quite so luxurious, perhaps, but equally comfortable. Spinal boards covered in baize are a novelty and will be found an invaluable adjunct to the schoolroom. The combination couch, lounge, and easy chair, as its name implies, is of protean shape, and is so handy for travelling that it is in large demand. Five guineas is not an excessive price when perfection of finish and detail is taken into account. It possesses the further merit of being ornamental as well as useful. The Merlin chairs are another feature to which we desire to draw the attention of our readers. They are self-propelling, and invaluable to the helpless and those who are unable to walk without assistance. We can speak from experience of the excellence both for in stitution as well as home purposes of the commode chair. When not used as a commode it makes a most comfortable armchair, and thus serves a twofold purpose. It is very strong yet light, so that there is no difficulty in moving it about, and it will stand any amount of hard wear. Admirable also are the bed-rests both in appearance and construc-

Bed Table and Desk.

tion. One with arms is particularly comfortable and much less likely to cause fatigue to the patient, who is thus enabled to support himself on his elbows should there be any tendency to slip off the pillows or down the bed. In conclusion, we advise all those who are interested in rendering the lot of the sick a less trying one to call and examine for themselves the ingenious devices that Messrs. Farmer, Lane, and Co. have brought to such perfection for the benefit of suffering humanity.

### Professional Pin-cushions.

It is usually the new probationer who is conspicuous for the completeness of her chatelaine, and who possesses an ingenuity in devising novelties in pin-cushions which, were such brain power and invention concentrated on her work, would go far towards preventing her staff-nurse from so frequently wearing a worried look. The staff-nurse, however, does not herself invariably despise a novel and original pin-cushion—especially when it is presented to her—idea and everything found. So varied are the shapes which pin-cushions assume that it may be interesting to note some of the novelties that now adorn the chatelaines of enterprising hospital pro's and their "staffs."

From the point of view of the inventor of new designs in pin-cushions much of the charm has vanished when the invention is copied. To be the wearer of a pin-cushion absolutely unique in the annals of ward history is to the new probationer almost as great glory as it is to the medical student to be chosen captain of his hospital cricket team.

A pretty and suggestive form of pin-cushion is when it takes the shape of a padded splint. Some varieties are extremely complicated; others are merely after the type of simple fracture splints, while more ambitious workers carry theirs up to the most complex splint appliance known to modern surgery. The surface may be plain or elaborately quilted; the object aimed at in either case is to make them look as professionally accurate as possible. A little piece of pink silk may be used to simulate jaconet, for the latter is too hard and unyielding to allow of the successful sticking in of pins.

Another pin-cushion savouring of the profession is a miniature roll of white water bandage. This kind of pin-cushion is evanescent and fleeting since it soon soils and the edges speedily fray. One probationer specially skilful at needlework evolved a pin-cushion as a present to "Sister," which is regarded as a triumph of the art of pin-cushion making. She fashioned a Liliputian stethoscope from cardboard, lightly wadded it, and covered it with brown velveteen. It really was most workmanlike, and when hung on the chatelaine looked quite prepared to do its lawful duty in the auscultation of dolls. Some nurses fasten their R.B.N.A. badges on to flat pin-cushions shaped to fit, and thus they are able to wear the medals of their association turned to practical account. Various hospital badges and medals are utilised in the same way, so that the wearer cannot—if she use her medal for the housing of pins—be suspected of any undue desire for personal ornamentation. A tiny imitation of a surgical syringe is another novelty in the pin-cushion department, but it needs some skilful architecture and shaping so as to preserve the original design. Novices with the needle should not attempt anything so ambitious as this. It is wiser for amateurs to keep to a more easily attained method of pin disposal.

Some nurses dispense altogether with pin-cushions and use instead a silver matchbox; others procure a common metal box closing with a spring, and cover it with plush or other soft material so as to prevent its noisy "jingling" as their owners move.

A pretty pattern of pin-cushion is one made in imitation of a rubber hot water bag. Given a modicum of inventiveness a glance round a hospital ward will carry with it many suggestions and variations of "professional" looking pin-cushions for those nurses who wish to attain a scientific completeness in the hangings of their chatelaines.

### Where to Go.

The Distressed Gentlefolks' Aid Association (Patroness, the Princess Christian).—A performance of "Captain Swift" will be given on Saturday, the 15th inst., at 8 p.m., at the Bijou Theatre, Archer Street, Westbourne Grove. Tickets, price 1s., 2s., 3s., and 5s., may be obtained from the Hon. Secretary, 75, Brook Green, W. Prince and Princess of Saxe-Weimar, the Duke and Duchess of Portland, and other influential ladies and gentlemen have given their patronage.

The Society for Promoting Female Welfare.—The annual united sale of work for the societies in connection with the above will be held in the Royal Albert Hall on April 26th and 27th. The Countess of Leven and Melville will open it at 12-30, and the charges of admission will be 2s. 6d. on the first day and 1s. on the second. The following are the societies benefited:—The Y.W.C.A. Home of Rest, Worthing; the Soldiers' Wives' Aid Society, Aldershot; the Chinese Bible Mission; the Palestine and Lebanon Nurses' Mission; St. Mary's Training Home; the Widows' Sewing Class, Mildmay; the Royal Home for Ladies; the Azilo, Bordighera; the Irish Ladies' Work Society; the Luther Memorial Home; Dr. Barnardo's Cottage Homes for Girls; the Y.W.C.A. Institutes, Bournemouth; the Church of England Zenana Missionary Society; the Mary Wardell Convalescent Home for Scarlet Fever; the Girls' Orphanage, Belleville, Paris; the Evangelical Girls' School, Figueras; the Cripples' Home; the Princess Mary Village Homes; the Illuminated Text Mission; Scott House, Hitchin; the Friends of Armenia; the Phœnix Blind Home; the British Syrian Schools; the Indigent Blind Visiting Society; the Cavendish Home, Hampstead.

### Appointments.

Jaffray Branch Hospital, near Birmingham.—On the 7th inst., Miss Rose Pauline de Chastelaine was appointed Matron of the above. She was trained at King's College Hospital, London, from 1891 to 1893; and for the two succeeding years worked at the new Hospital for Women, London. She then became sister at the Suffolk General Hospital, Bury St. Edmunds, and superintendent of the Union Infirmary, Old Down, Bath, which post she now relinquishes.

Aston Union Workhouse, Gravelly Hill, near Birmingham.—On March 28th Miss Gertrude M. Oldacre was appointed superintendent nurse here. She was trained at Addenbrook's Hospital, Cambridge, and was afterwards night superintendent at Chorlton Union Infirmary, Manchester.

Keighley and Bingley Joint Hospital.—Miss Florence Appleby was appointed matron of this Hospital on the 5th instant. She was trained at the London Hospital, and has worked at the City Hospital, Birmingham, and as District nurse at Bingley and at London.

### Typical Patients.

### THE REFRACTORY PATIENT.

He occupied No. 8 bed in Montgomery Ward. He was a "trial"—nurse said so, and even the sister said so. He was about forty years of age, a bachelor, a short-tempered and sharp-featured fellow, who was an inmate of the hospital only under protest. He arrived on a hired ambulance wheeled by his landlord and his landlord's son. They could no longer "stomach him," as they pointedly styled their feelings in the matter. He had been a lodger with them for several years. His one good quality, according to them, was his habit of paying his rent regularly when in work. But as his illness had taken a serious turn, and as there was but little or no prospect of work being within his reach for a considerable time, the landlord suddenly arrived at the conclusion that he would be better attended to in hospital.

He would insist on eating his bread and butter with the buttered side underneath. He used to say that the butter went further when taken in that way. The Sister, of course, thought that it did not, and that he made the bed-clothing very dirty by putting the buttered side of his bread down on his pillow or sheet while taking up and drinking from his mug of tea. On one or two occasions a nurse was specially told off to sit beside him and see that he did not indulge in his dirty practice, but unless some physical and, possibly, damaging force was used there was no getting over him. He would argue, and, like all foolish persons, he was capable of putting a question that a wise person could not always satisfactorily answer. He would not try to go to sleep at eight p.m. He used to say that his time was ten, and ten it was going to be till the end of the chapter. He was threatened by the house physician, who told him that if he would persist in talking and preventing obedient patients from going to sleep he should be placed in an isolation ward, where he would have nothing to look out upon but bare walls, and where he could talk and swear and shout the whole night through without discomfort arising for anyone but himself. But he said that that was just what he would himself prefer. "I shall have to have a nurse all to myself then, eh, doctor? I 'spose I could get waited on properly then?"

When the chaplain came, and a Psalm was quietly sung in the ward, he would read the verses aloud during the singing. He declared he did not hold with singing the Psalms. "I 'aint agoin' to be bamboozled into your 'igh church ways, not me! Don't you think as the poor don't know what's right." The chaplain would kindly point out that there were no very critical cases in the ward, and that the majority of the inmates were in favour of the Psalms being sung; would he, therefore, oblige by remaining quite quiet, if his conscience would not allow him to take an active part in the worship. But no; he continued to create a disturbance by reading aloud in his deep harsh voice.

He had a rooted objection to the students. However gentle or however firm they were he would not have them near him. He threw articles at their heads; he pulled all the bed-clothing from his poor thin body and attempted to chase the offenders away.

The steward was summoned and talked seriously to him. "If you carry on in this manner, you know, we shall have to pack you off to the Union infirmary. We can't have all the rules set aside by one obstinate patient." He replied, "Oh, no; yer won't send me away. I know yer won't, because I 'eard one of the doctors say as how my case was a very interestin' one, and that he was very anxious to see 'ow I got on under the new treatment. Wait till I gets out again. I'll tell the public what their money is done with; you trust me for that. You're a d——d set of experimenters, that's wot you are, every d——d one of yer."

As a matter of fact, his case was not the one he heard referred to at all. He was labouring under a delusion, as so many of us do when poorly. He gradually lost strength and died peacefully. His case was well-nigh hopeless from the first. He had no friends, and the few flowers that were placed upon the plain coffin were put there by the kind hands of the chaplain, to whom, when alive, he was so vigorously opposed.

### Minor Appointments.

Chelsea Infirmary.—Miss Ellen Copland Crichton, who was trained at the Chelsea and the Edinburgh Royal Infirmaries, has been appointed Night Superintendent of the above. Miss Crichton has since been on the staff of the Nurses' Co-operation, 8, New Cavendish Street, and holds excellent testimonials.

Park Hospital, Hither Green, S.E.—Miss Alice Maud Smythe, charge nurse of the above, has been promoted to be Night Superintendent. She was trained at the London Hospital, and has been charge nurse at the Taunton and Somerset Hospital, sister at the City Hospital, Leeds, and charge nurse at the North Eastern Hospital.

Sheffield Union Children's Hospital.—Miss Lizzie Hutchinson was appointed Charge Nurse here on April 5th. She was trained at Salford Union Infirmary, and has been superintendent nurse, Holborn Union School Infirmary, Mitcham.

Derby County Asylum.—Miss Mary Campbell has been appointed Chief Nurse of this asylum. She has been nurse for six years at the Lancaster County Asylum, for three years at the West Derby Union, and at the Mill Road Infirmary, Liverpool.

Patricroft Workhouse, Barton-upon-Irwell.—On the 5th inst. Miss Letitia Middleton, who was trained at Salford Union Infirmary, was appointed Charge Nurse of this institution. Her previous appointments have been at Haslingden Union Infirmary and at the Brook Fever Hospital.

Huddersfield Infirmary.—On March 24th Miss Ada Mander was appointed Charge Nurse of this infirmary. She was trained here, and afterwards became charge nurse at the County Infirmary, Downpatrick, and charge nurse of the theatre at the Banbury Infirmary.

Bolton Infirmary.—Miss E. M. Crawford, who was trained at the Leeds Infirmary for three years, was appointed Night Superintendent here on March 9th. She was for two years sister of the male and female surgical wards and theatre at the District Hospital, Grimsby.

Halstead Union Workhouse.—On March 24th Miss Harriet Squibb was appointed head nurse of this workhouse. She was trained at the Halifax Union Hospital, and has been employed in the Halifax and York Workhouses.

Victoria Hospital, Blackpool.—On the 6th inst. Miss Gertrude Willmott was appointed Sister at the above. She was trained at the Leeds General Infirmary, and was afterwards nurse at Stratford-on-Avon Hospital.

Colchester Workhouse Infirmary.—Miss Rose Turner was on March 21st appointed Superintendent-nurse of the above. She was trained and afterwards charge nurse at Leeds Union Infirmary.

Blandford Cottage Hospital.—On April 10th Miss E. J. Neve, who was trained at the London Hospital, E., was appointed Matron here. Niss Neve has been matron of the Cottage Hospital, Enfield.

### Everybody's Opinion.

[Correspondence on all subjects is invited, but we cannot in any way be responsible for the opinions expressed by our correspondents. No communication can be entertained if the name and address of the correspondent is not given, as a guarantee of good faith but not necessarily for publication, or unless one side of the paper only is written on.]

### POISON BOTTLES.

"Matron" writes: I see in last week's Hospital a nurse inquiring about bottles for poisons so that the use for which they are intended could be detected. A short time ago a friend of mine gave me a bottle intended for poisons, and to the cork was attached a little bell. You could not remove the cork without ringing the bell. I thought perhaps other nurses might like to hear about it, for I think it a very good idea. I might add the bottle I had given to me came from a chemist at Salisbury, but I have no doubt they could be ordered through any chemist.

### ENLARGEMENT OF "THE MIRROR."

Miss Mary S. Crossland, writing from the Nest, Walcott, Stalham, Norwich, says: The enlargement of the "Nursing Mirror," in consequence of its increased popularity, seems to me to afford a good opportunity to offer my testimony to the usefulness of the paper and the interesting matter it contains —interesting to all busy hospital nurses; still more, I think, to those working in isolated places, and equally so to those who, in consequence of advanced age or sickness, have left the work, though not given up their interest in the workers. For twenty-one years I was sister in charge of the Nightingale Training School for Nurses, St. Thomas' Hospital. During those years some hundreds of probationers passed under my care. It is impossible for me to keep in touch with more than a few of them. The "Nursing Mirror" comes to my aid as a connecting link. Every week in my quiet country home when I receive the paper I at once look to the appointments major and minor, and I often have the pleasure of seeing a name I know. I write and congratulate the owner on her appointment, and always receive a kind reply. With your permission, I also wish to express my warm appreciation of the benefits accruing to members of the Royal National Pension Fund, of which I early became a member, No. 139. I have encouraged many nurses to join, saying, "I only wish there had been such an institution when I commenced my career."

### A CRUSADE OF CLEANLINESS.

"S. E. R." writes: Having read your article on this subject in The Hospital of the 8th inst., I should like to make a few remarks, if I may be allowed a little space in your columns. During the last six months I have seen some of the great good a nurse visiting the London schools can effect, and has already effected. It will, I am sure, be a pity if the London School Nursing Society is not soon well established in all the schools in London and its surrounding districts. But I am afraid that such a result will be a long time in coming, unless steps are taken to inform all the head masters and mistresses, officially, that such work is to be done in their schools. It is most discouraging for a nurse to go to a school with cheerfulness and alacrity, intending earnestly to do all the good she can, to be met by the master, who says, "he knows nothing about the society," and considers it an insult to his school to suggest that his children want any looking after as to their personal cleanliness, &c., and that he must decline to receive anyone until he has had official notice from headquarters. This has happened to me in three schools where the children were of the poorest and dirtiest class. If masters of these schools had received notice in an official way, the nurse would have been treated with courtesy at any rate. I am now working in a very poor district where the masters strongly oppose the scheme of the London School Nursing Society, but I have visited some of the children in their homes, and I find that the mothers and children are glad of this nursing help. Yet some of the masters will not allow them to obtain it. Nurses cannot, and will not, work if they are met in this manner. There are masters and mistresses who take a lively interest in the physical well-being and cleanliness of the children, and who welcome the visiting nurse with pleasure, and help her in every possible way. It is in their schools that the work has been productive of lasting good. The indifference, to say nothing of the determined opposition, of many of the teachers must be first overcome, and then the society will soon make satisfactory progress.

### For Reading to the Sick.

### "IT IS MORE BLESSED TO GIVE THAN TO RECEIVE."

### Verses.

Blessing she is; God made her so;

And deeds of weekday holiness

Fall from her noiseless as the snow;

Nor hath she ever chanced to know

That ought were easier than to bless. —*Lowell.*

Beloved, yield thy time to God, for He
Will make eternity thy recompense;
Give all thy substance for His love, and be
Beatified past Earth's experience. . . .
Shall setting day win day that will not set?
Poor price wert thou to spend thyself for Christ,
Had not His wealth Thy poverty sufficed;
Yet, since He makes His garden of thy clod,
Water thy lily, rose, or violet,
And offer up thy sweetness unto God.
—*Christina Rossetti.*

### Reading.

Who, seeking for himself alone, ever entered heaven? In blessing we are blest.—*C. Seymour.*

Strew human life with flowers! Save every hour for the sunshine! Exalt your souls! Widen the sympathies of your hearts! make joy real now to those you love!—*Richard Jefferies.*

The appearance of separation or isolation in anything, and of self-dependence, is an appearance of imperfection; and all appearances of connection and brotherhood are pleasant and right, both as significant of perfection in the things united and as typical of that unity which we attribute to God. The unity of spirits is partly in their sympathy, and partly in their giving and taking, and always in their love; and these are their delight and their strength; for their strength is in co-working and army fellowship, and their delight is in the giving and receiving of alternate and perpetual good, their inseparable dependency on each other's being, and their essential and perfect depending on their Creator's. And so the unity of earthly creatures is their power and their peace; not like the dead and cold peace of undisturbed stones and solitary mountains; but the living peace of trust, and the living power of sympathy; of hands that hold each other—and are still.—*Ruskin.*

It takes us all a long time and a sharp discipline to learn that he who would keep his life must first lose it, and that to empty oneself is the sure way to be filled. The heart of man is so constituted that its fulness comes of spending. When we serve—we rule. When we give—we have. When we surrender ourselves, we are victors. We are most ourselves when we lose sight of ourselves. He is the most certain to have his own way, and to find pleasure in it, who deliberately chooses to resign his preference in favour of others. We know not what we are or might be. As the seed has within it, so men have within them—angels.—*Newman.*

Is not making others happy the best happiness? To illuminate for an instant the depths of a deep soul . . . is to me a blessing and a precious privilege. There is a sort of religious joy in helping to renew the strength and courage of noble minds. We are surprised to find ourselves the possessors of a power of which we are not worthy, and we long to exercise it purely and seriously.—*Amiel.*

### Our Convalescent Fund.

We acknowledge with many thanks S. A. M.'s (Policy 680) kind gift of 5s. to the above fund.

### Travel Notes.

By Our Travelling Correspondent.

### XVIII.—THE VOYAGE UP THE NILE.

### Different Modes of Making It.

There are many ways of making this delightful excursion, but all are expensive. You can go by Cook's or Gaze's steamers, occupying either three or four weeks on the journey, according to convenience. Then there is a way of going by the mail steamers, time occupied to Assouan and back fourteen days; and yet another plan, by which you employ the train to Nag Hamadeh, and take to the mail steamer there. These last are hurried ways, but it is well to know of them, for professional men, and, indeed, many others, have often but a very limited time in which to take their holiday, and it is better to make the Nile voyage in a somewhat perfunctory manner than not at all. There is a way less costly than any of the foregoing; it is to take the train to Nag Hamadeh, and then to secure a passage in a feluka, that is to say, in a native sailing-boat used for the conveyance of provisions. You can understand that accommodation is not luxurious, but if you are strong and of an adventurous disposition it would be a most interesting experience. N.B.—Ladies alone must not attempt it, even in these emancipated days. Then comes the journey by dhahabîyeh, taking from two to three months, the most agreeable, but also the most expensive, of all the modes of ascending the Nile. Now let us consider the varying cost of these different means of transit.

### Cook's and Gaze's Steamers.

As this is the most popular way of making the journey we will consider it first. Cook's steamers go once a week from November to the middle of March, taking three weeks there and back. Fares £50 or £60 if the best cabins are taken, suitable for invalids. This sum includes everything, food (except wine), and all unavoidable expenses, such as donkeys, guides, boats, &c., on small expeditions. The Government tax is not included. It amounts to £1 0s. 6d., and is levied upon all visitors towards the "maintenance of the monuments." Messrs. Cook or Gaze will arrange this for you. For luggage you may take 200 lbs., but the size of trunks must not be more than two cubic mètres. Messrs. Gaze arrange Nile tours on still cheaper lines, taking 25 days on the trip, fare £42. This is by the steamers of the Nile Navigation Company. By the Anglo-American line they make the journey in 20 days, fare £35 to £45, and they arrange an eight days' trip beyond Assouan to Wady Halfa and back for those who have booked through from Cairo for £20. Undoubtedly these steamer lines conducted by Cook and Gaze are the most generally convenient.

### The Dhahabîyeh Journey.

Incomparably this is the more agreeable way, but it has its disadvantages, primarily the great expense, and secondarily the time that must necessarily be spent. Few people have the leisure even if they have the money, consequently the steamboat mode is the most popular. Still one must admit that, given a sufficiency of time and money, the dhahabîyeh is the ideal way of transit. If the party consists of four to six persons the expense is greatly reduced, and in planning such a trip it is usual to arrange for that number or more. It is best to engage one of Cook's or Gaze's dhahabîyehs. Gaze's are a trifle cheaper, but in the end I think there is not much real difference. I will quote Cook's prices. For a party of five persons they charge for two months £590, for a larger party less by comparison, and for a smaller more. They have a steam dhahabîyeh, for which the cost per month is £400 for four persons, and to those to whom time is of great importance and money no object it is as well to know of it. There are private dhahabîyehs to be had in Cairo, and they are much cheaper, but I do not advise them. The chartering is such a troublesome affair, involving the drawing-up of divers agreements, the engaging of Dragoman and Ries (Captain), &c., and probably in the end you find you have spent more money, had less comfort, and lost both time and temper.

### By Rail and Mail Steamer.

By this route considerable time is saved and some expense, but railway travelling in Egypt is not agreeable, it must be remembered. You take the train to Nag Hamadeh and then go on by the mail steamers organised by Messrs. Cook. The railway journey occupies about fourteen hours. The return can be made in the same way, or the entire distance can be taken by mail steamer.

### By Feluka all the Way.

This last would be my choice if time and health permitted, but I hardly think many of my readers would share my taste; still I will tell you how it is managed in case the spirit moves some adventurous spirit to try. Felukas are to be hired at Cairo for ten francs per day, and only have cabin accommodation for one or two. You would need to engage an Arab servant who could cook, at wages of three francs a day, and you must lay in provisions for your lengthy voyage. In these stores you must include tea, coffee, tinned meats, soups, fish, vegetables, and fruit, a large store of flour, as bread must be baked; hams, bacon, rice, macaroni, arrowroot, sultanas, raisins, these latter for puddings and cakes to be made on baking days, butter in tubs or tins, sugar, salt, soda, soap, insect powder (very important), an oil cooking stove, a reading lamp, paraffin oil, matches, plenty of blankets, cups, plates, knives and forks, &c. It would be well to inspect the feluka carefully first, so as not to omit necessaries. I feel sure no toilet glass will be discernible, and I should judge the washing apparatus (if any) would leave much to be desired. I had two friends some years ago who made the journey in this way, and their experiences were most amusing, but probably, now that swarms of tourists people the Nile route, even the felukas have risen more or less to the occasion. I could say much about the glorious sights to be met with on the Nile, but must not embark upon so vast a field of interest. With the good guide books now to be had, every traveller can find out for himself what is likely to prove most interesting to him individually. Sketchers and photographers—and almost everyone is either one or the other in these days—will find their pleasure greatly enhanced. I respectfully submit to the notice of the former the difficulties of rendering the luminous atmospheric effects of Egypt; may they be more fortunate than I was.

---

### Hints to Those Meaning to Sketch.

Sketching materials are always heavy, and there is an uncompromising knobbiness and development of corners exasperating to the packer. I find it wise to have a basket, square and covered with waterproof, in which to keep all artistic impedimenta. By this arrangement, if you are stopping only one night in a place, it is not necessary to disarrange the general trunk in a wild and hurried hunt for sketching materials. The basket may be of very modest dimensions, the exact length and width of your sketching block or board, and deep enough to accommodate the colour box, water bottle, brushes, bottles, &c., under the block. The easel and umbrella, now made to fold into the most convenient length, may be strapped outside or put with the wraps and other sticks and parasols. Now may I tell you of what I find the most convenient form of sketching apparatus and one that has visited all countries with me. I asked the carpenter to make me a common deal frame two and a half inches wide and about sixteen inches by eleven and a half, the size to take one quarter of a sheet of Whatman's paper. I then took four sheets of this same Whatman's paper (it may be rough or smooth, according to fancy), one shilling per sheet, and divided them each in four, which gave me sixteen pieces. I thoroughly wetted these with a clean sponge, allowing them to lie soaking on a clean wet towel for two hours, then nailed eight pieces on each side of the frame, putting in ordinary small tin-tacks at one and a half inches apart. When this is dry it makes a firm, delightful surface to paint on, and there is the advantage of having two sketches going at once. As each picture is finished slip a penknife round inside the nails and cut off. To avoid one sketch becoming dirty whilst the other one is being worked on, I have a piece of calico large enough to cover both sides nailed along the upper side, and it can thus be secured over one surface or both with drawing pins. If you like to vary the size of your sketches, you might have two small frames made exactly half the size of the first; treat them in the same way and they will exactly cover the large one in your basket. Take a reserve of paper, for it is very dear on the Continent, and generally of inferior quality, except in large cities. Do not burden yourself with an infinite variety of colours. One artist I knew, who created the most charming pictures, limited himself to cobalt, light red, and yellow ochre. I admit it takes a genius to succeed with this meagre supply, but one may humbly follow on somewhat the same lines. I have always noticed that the more incompetent the artist the more elegant are his or her appliances.

### FIFTEEN DAYS' EXCURSION TO THE ITALIAN LAKES FOR £11 11s.

IN the travel advertisements you will see a notice of this cheap and popular excursion, which starts on Saturday, May 27th. Last time several of our nurses joined, and greatly appreciated the trip, with the complete change of scene, climate, people, and indeed everything else which it afforded. It would be quite impossible, travelling independently, to spend so small an amount, for the return ticket to Como alone is £8, whereas in this programme one sees all the most beautiful lakes of North Italy, has one's hotel expenses and meals on the journeys all paid for £11 11s. There are no extras except such as one may choose to make for oneself by varying the programme or extending the stay. I think anyone would find it a charming fortnight's change, and if they will ask me through "Travel Notes and Queries" concerning costume and necessaries I shall be pleased to reply. I say this because I noticed on the only tour of this kind in which I took part so many people had burdened themselves with articles never used or needed, and had omitted absolute essentials, especially in the matter of clothing. The receptacles for one's possessions should be as light as possible, and of convenient size and shape.

*(For Travel Advertisements see Page* xliii.*)*

### Resignations.

MISS SMITH, the matron at the Eston Sanatorium, under the Eston Urban District Council, has resigned her appointment in order to take up her duties as Matron of the White Hoe Hospital, Douglas, Isle of Man. She was trained for three years at the Parish Infirmary, Brownlow Hill, Liverpool. Since then she has been charge-nurse at the City Hospital, North Liverpool; charge-nurse at the Eastern Hospital, Homerton, London; and for the past five and a-quarter years matron at the Eston Sanatorium.

### Notes and Queries.

The contents of the Editor's Letter-box have now reached such unwieldy proportions that it has become necessary to establish a hard and fast rule regarding Answers to Correspondents. In future, all questions requiring replies will continue to be answered in this column without any fee. If an answer is required by letter, a fee of half-a-crown must be enclosed with the note containing the enquiry. We are always pleased to help our numerous correspondents to the fullest extent, and we can trust them to sympathise in the overwhelming amount of writing which makes the new rules a necessity.

Every communication must be accompanied by the writer's name and address, otherwise it will receive no attention.

*Strength of Staff.*

(18) Can you please tell me the average number of patients to each nurse in the principal Poor Law infirmaries? 2. What "staff" would you consider necessary in an infirmary containing 250 beds, the majority of patients being weak and helpless?—*Superintendent.*

1. There is no general rule, and the number varies. The best plan, however, is to refer to concrete examples. According to a Parliamentary return made two or three years ago, giving the number of persons occupying wards for the sick and the number of paid officers acting as nurses, it appears that the following were the proportions existing between the number of "sick and bed-ridden" in the various institutions mentioned, and the number of "paid officers acting as nurses" in the same places: Paddington, 178, 26; Kensington, 565, 60; Fulham, 443, 46; Chelsea, 298, 35; St. George's, 615, 50; St. Marylebone, 571, 68; St. Pancras, 449, 58; &c. This will serve to show something as to the numbers in some of the metropolitan separate infirmaries under the Poor Law. 2. It is quite impossible for anyone not conversant with all the circumstances of the work to say how many nurses are required to 250 beds.

*Certificate.*

(19) Can you kindly tell me if it is possible to train in Glasgow for a midwife's certificate without living in any hospital or institute, and what the fees are likely to be?—*A. C.*

You could make arrangements with a local midwife to prepare you for the London Obstetrical Society's certificate. The fee would probably range from £5 5s., without examination expenses.

*Sanitary Inspector.*

(20) Would you kindly tell me the best way to become a sanitary inspector?—*Evelyn.*

Apply to the Secretary, the Sanitary Institute, Margaret Street, London, W., for information.

*Disinfection.*

(21) Can you kindly inform me if there is any place near London where nurses would be taken after being at an infectious case before going to another; or at the seaside not far from London?—*A.F.C.*

Facilities for disinfection are offered to nurses at the Nurses' Hostel, Francis Street, N.W.

*Books.*

(22) I want to get a good book on general nursing, and should be glad if you could advise me. Do you think "A Handbook for Nurses," by Watson, would be suitable? and could you also tell me of a good book on fevers?—*M. H.*

Dr. Watson's Handbook for Nurses will shortly be published by the Scientific Press. In regard to fevers, "Fevers and Infectious Diseases," by the same publishers, can be recommended. "Nursing," by Isabel Adams Hampton (7s. 6d.); or "Nursing: Its Theory and Practice," by Percy G. Lewis, 3s. 6d. (the Scientific Press) are regarded as standard works on the subject.

*Royal National Pension Fund.*

(23) Would you kindly let me know through THE HOSPITAL where to write for particulars, &c., of "the Nurses' Royal Pension Fund," and if you think the same a good one?—*B. B.*

The Secretary, the Royal National Pension Fund, 28, Finsbury Pavement, E.C. It offers nurses unique advantages, as you will see by studying the prospectus. There is no other society that gives bonuses to subscribers for an annuity

*Bicycle Repairs.*

(24) I am a district nurse engaged in a rural district, and, having to travel long distances, my committee have supplied me with a bicycle, and now the question arises who ought to pay for all necessary repairs caused through damages during my use of it whilst doing my duty. I or the committee?—*M. J. S.*

The committee, certainly.

*A Children's Hospital.*

(25) I am anxious to train in a children's hospital, can you advise me as to which one is best? I am 19, tall, strong, and healthy.—*An Anxious One.*

Send 2s. to the Scientific Press for "The Nursing Profession: Where and How to Train." You will find full particulars in it.

*Floor Staining.*

(26) "S. M." would be much obliged for advice on the cheapest and best paint (brown) and varnish preparation for floors of large school. The ordinary staining—bees-wax polishing—unsatisfactory, dangerous, and the colour disappears with fumigation (sulphur).

Permanganate of potash dyes the boards a good brown, that is if they are clean and free from wax and grease. The colour can be regulated by the strength of the solution used. A coat of glue size, and another of oak varnish afterwards, gives good results.

*Vacant Matronships.*

(27) Will you kindly tell me how and where I could hear of any possible vacant matronship in an orphanage, industrial school, or other institution for children in or near London?—*Cecil.*

Consult advertisements in the County Council and Poor Law Journals.

## Figures and Tables

**Figure f1:**
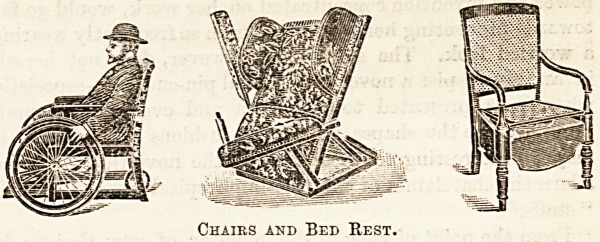


**Figure f2:**